# Formulation and Evaluation of *In-vitro* Characterization of Gastic-Mucoadhesive Microparticles/Discs Containing Metformin Hydrochloride

**Published:** 2014

**Authors:** Fatemeh Khonsari, Parvin Zakeri-Milani, Mitra Jelvehgari

**Affiliations:** a *Student Research committee, Tabriz University of Medical Sciences, Tabriz, Iran.*; b *Faculty of Pharmacy, Tabriz University of Medical Sciences, Tabriz, Iran. *; c *Liver and Gastrointestinal Diseases Research Center, Tabriz University of Medical Sciences, Tabriz, Iran. *; d *Drug Applied Research Center, Tabriz University of Medical Sciences, Tabriz, Iran.*

**Keywords:** Gastric-mucoadhesive, Microparticle, Metformin hydrochloride, Emulsion solvent evaporation technique, Ethylcellulose, Carbomer 934p

## Abstract

The present study involves preparation and evaluation of gastric-mucoadhesive microparticles with Metformin Hydrochloride as model drug for prolongation of gastric residence time. The microparticles were prepared by the emulsification solvent evaporation technique using polymers of Carbomer 934p (CP) and Ethylcellulose (EC).

The microparticles were prepared by emulsion solvent evaporation method (O_1_/O_2_). Disc formulations were prepared by direct compression technique from microparticles. In the current study, gastric-mucoadhesive microparticles with different polymers ratios (CP:EC) were prepared and were characterized by encapsulation efficiency, particle size, flowability, mucoadhesive property and drug release studies.

The best polymers ratio was 1:3 (F_2_) with Carbomer 934p (as mucoadhesive polymer) and ethylcellulose (as retardant polymer), respectively. The production yield microparticles F_2_ showed 98.80%, mean particle size 933.25 µm and loading efficiency %98.44. The results were found that microparticle discs prepared had slower release than microparticles (p > o.o5). The microparticles exhibited very good percentage of mucoadhesion and flowability properties. The release of drug was prolonged to 8 h (71.65-82.22%) when incorporated into mucoadhesive microparticles. The poor bioavailability of metformine is attributed to short retention of its dosage form at the absorption sites (in upper gastrointestinal tract). The results of mucoadhesion study showed better retention of metformine microparticles (8 h) in duodenal and jejunum regions of intestine (F1, 1:2 ratio of CP:EC).

Therefore, it may be concluded that drug loaded gastric-mucoadhesive microparticles are a suitable delivery system for metformin hydrochloride, and may be used for effective management of NIDDM (Non Insulin Dependent Diabetes Mellitus).

## Introduction

Solvent evaporation technique is comparatively simple and has been applied for the encapsulation of a number of pharmaceuticals. It involves two steps such as the preparation of emulsion containing polymer and drug with a supplementary medium in which the drug and polymer cannot dissolve, followed by the complete removal of solvent. Stomach-specific mucoadhesive as a controlled drug delivery system have been developed to increase gastric retention time of the dosage forms (1) Gastric mucoadhesive drug delivery has been suitable for some drugs with short transit time in upper segment of stomach and low bioavailability. These drugs characterized by narrow absorption window in the superior part of gasterointestinal (GI) tract as stomach and small intestine. The long gastro-retentivity time of drug is necessary in this part of GI tract (6-8 h). Then, the drug has left the upper of GI tract and it is released in non-absorbing distal segments of the GI tract. Gastro-mucoadhesive drug delivery is improved absorption properties of drugs with narrow absorption window as large surface are, retain in upper of GI and released the drug there in a controlled and prolonged, in comparison to the colon. This state of drug delivery, could be supplied continuously to its absorption sites in the upper GI tract and may perform many benefit containing improved bioavailability, reduction of dose size, decrease frequency of drug administration and improve the patient compliance (1). Several theories have been put forward to explain the mechanism of polymer–mucus interactions that lead to mucoadhesion. To start with, the sequential events that occur during bioadhesion include an intimate contact between the bioadhesive polymer and the biological tissue due to proper wetting of the bioadhesive surface and swelling of the bioadhesive. Following this is the penetration of the bioadhesive into the tissue crevices, interpenetration between the mucoadhesive polymer chains and those of the mucus. Subsequently low chemical bonds can become operative. Mucoadhesive bond is formed will depend on the nature of the mucous membrane and mucoadhesive material, the type of formulation, the attachment process and the subsequent environment of the bond. The wetting theory considers surface and interfacial energies and is primarily applied to liquid systems. This theory proposes that as a prerequisite for the development of adhesion the liquid should have the ability to spread spontaneously onto a surface. The adsorption theory proposes that hydrogen bonding and van der Waals’ forces are the main contributors to the adhesive interaction. Hydration of the polymer plays a very important role in bioadhesion. There is a critical degree of hydration required for optimum bioadhesion. If there is incomplete hydration, the active adhesion sites are not completely liberated and available for interaction. On the other hand, an excessive amount of water weakens the adhesive bond as a result of an over extension of the hydrogen bonds. During hydration, there is a dissociation of hydrogen bonds of the polymer chains. The polymer–water interaction becomes greater than the polymer-polymer interaction, thereby making the polymer chains available for mucus penetration. Some factors affect on mucoadhesion including the physicochemical properties (as concentration of polymer, pH of polymer-substance interface, swelling of polymer) (1).

Ethyl cellulose (EC) is a non-toxic, stable, compressible, inert, hydrophobic polymer that has been widely used to prepare pharmaceutical dosage forms. This polymer is often used as a rate-controlling membrane to modulate the drug release from dosage forms with organic or aqueous coating techniques (2-4) but few references have focused on the use of EC as directly compressible excipient (2,5-7).

As a mucoadhesive polymer, Carbomer 934P (CP) has been investigated extensively by the pharmaceutical researchers in the preparation of mucoadhesive microspheres because of its good mucoadhesive properties and is not absorbed by body tissues and being totally safe for human oral consumption. It has high viscosity at the low concentration and low toxicity. The *in-vitro *experiment has proved CP having good bioadhesion with the gastrointestinal mucus (8).

Metformin hydrochloride (MH) is a widely used biguanide anti-diabetic drug for the management of non-insulin dependent diabetes mellitus. MH has been reported to control glucose level and improve lipid profile in type-II diabetics. MH may stop the growth of tumor cells by blocking some of the enzymes needed for cell growth. As MH affords a similar level of blood sugar control to insulin and sulfonylureas, it appears to decrease mortality primarily through decreasing heart attacks, strokes and other cardiovascular complications. MH is primarily absorbed from the small intestine. The extent of MH absorption is improved when the gastrointestinal motility is slowed (1). Therefore, the bioavailability of this drug even from aqueous solution or rapidly dissolving tablets is relatively low. A pharmacokinetic-pharmacodynamic rationale for development of MH controlled release formulations was established and concluded that clinical advantage could be obtained from gastro-retentive systems. Hence to optimize oral MH therapy, there is a need for development of MH tablets, which confine to specific site in the upper intestine. Mucoadhesive microspheres formulation is suitable for MH, therefore, the slow but complete drug release in the stomach is expected to increase bioavailability of the drug as well its perfect employment which may results to, lower dose and GI side effects (as less chance of dose dumping  (9,10).

In the present study, an attempt was made to develop oral mucoadhesive controlled release MH microparticles using EC and CP. Mucoadhesive drug delivery is a topic of interest in the design of drug delivery systems to prolong the residence time of the dosage form at the site of application or absorption and thereby facilitating intimate contact of dosage form thus improving and enhancing bioavailability.

## Experimental

*Materials*

Metformin hydrochloride was purchased from Mahban Chemical Company (Excir, Iran), carbopol 934P (B.F.G, USA), Ethyl Cellulose 48 cP (Sigma-Aldrich, USA), n-hexane, ethanol, Span 80, hydrochloric acid (Merck, Germany). All other chemicals used were of either laboratory or analytical grade.

*Methodology*

*Method for preparation of ethylcellulose and carbopol 934P microparticles*

**Figure 1:**
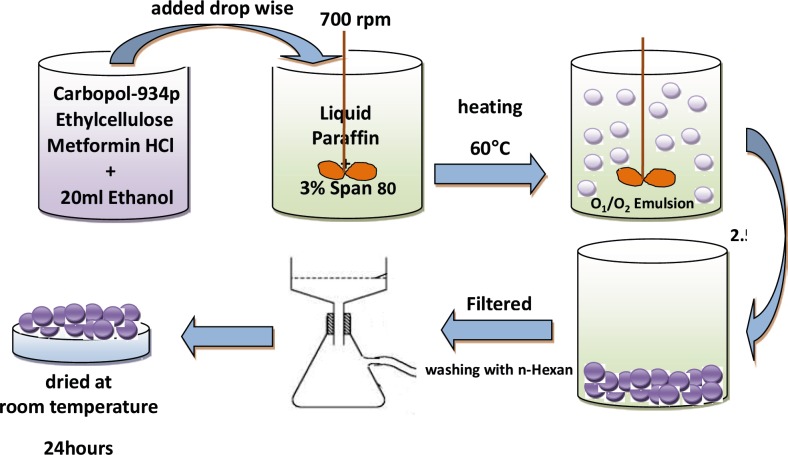
Schematic representation of the emulsification evaporation technique

Ethylcellulose (EC) and Carbopol 934P (CP) with three different CP/EC ratio (1:2, 1:3 and 1:4 w/w) were dissolved in 20 mL of ethanol using magnetic stirrer; weighed 500 mg of MH was added to the EC–CP solution under magnetic stirring (Table 1). Then the suspension was quickly injected using a 5 mL syringe into 125 mL of light liquid paraffin contained in a 250 mL beaker, which contains 3% (w/v) of Span 80, while stirring using a mechanical stirrer. Stirring rate was kept at 700 rpm to form an O_1_/O_2_ emulsion and was heated to 60 °C. Stirring was continued for 2.5 h at this temperature until ethanol removed completely and microspheres were formed. The hardened microparticles were collected by filtration and washed with three portions of 50 mL of n-hexane and air dried at room temperature for 24 h (Figure 1).

**Table 1:** Metformin Hydrochloride microparticle formulations prepared by emulsion solvent diffusion method (o_1_/o_2_).

**Formulations**	**Polymers (CP: EC) ratio**	**Emulsion (O**_1_ **/O**_2_ **)**					
		**Internal organic phase (O**_1_ **)**				**External organic phase (O**_2_ **)**	
		MH (mg)	Ethanol (mL)	Carbomer 934p (mg)	Ethylcellulose (mg)	Liquid paraffin (mL)	Span 80 (%w/v)
F_1_ F_2_ F_3_	1:2 1:3 1:4	500 500 500	20 20 20	225 225 225	450 675 900	125 125 125	3 3 3

* EC (Ethylcellulose), CP (Carbomer 934p) and (MH) Metformin Hydrochloride

*Determination of drug entrapments efficiency, drug loading, and yield*

Microparticles (100 mg) were weighed and crushed with mortar and pestle, then were suspended in 10 mL of 0.1 M HCl. After 24 h, the solution was filtered and the filtrate was diluted up to 100 mL with 0.1 M HCl. Next, 2 mL from this solution was picked up; this filtrate was diluted up to appropriate dilution (10 mL); and the drug concentration was measured spectrophotometrically (UV-160, Shimadzu, Japan) at 205 nm against 0.1 N HCI as a blank. The loading efficiency (%) was calculated according to the following equation:

Loading efficiency (%) = (actual drug content in microparticles/theoretical drug content) × 100

The production yield of the microparticles was determined by calculating accurately the initial weight of the raw materials and the last weight of the polymeric particles obtained to the initial weight of the raw materials. Each determination was performed in triplicate manner.

*Frequency distribution analysis*

Samples of microparticles was analyzed for frequency distribution with calibrated optical microscope; fitted with a stage and an ocular micrometer. Small quantities of microsphere were spread on a clean glass slide and the average size 60 particles, frequency distribution were determined in each batch.

*Differential Scanning Colorimetry (DSC)*

The physical state of drug in the microspheres was analyzed by Differential Scanning Calorimeter (Shimadzu, Japan). The thermo grams of the samples were obtained at a scanning rate of 10 °C/min conducted over a temperature range of 25-300 °C.

*Flowability characterization of microparticles*

*Angle of repose*

Angle of repose of different formulations was measured according to fixed funnel standing method. 

 θ = tan^-1^ *h* /* r*

Where θ is the angle of repose, *r* is the radius, and *h* is the height.

*Bulk and tapped densities*

Bulk and tapped densities were measured by using 10 mL of graduated cylinder. The sample poured in cylinder was tapped mechanically for 200 times, then tapped volume was noted down and bulk density and tapped density were calculated. Each experiment for micromeritic properties was performed in triplicate manner.

*Carr's index*

Compressibility index (Ci) or Carr's index value of microparticles was computed according to the following equation:

Carr’s index (%) = (Tapped density – bulk density) / Tapped density x 100

*Hausner's ratio*

Hausner's ratio of microparticles was determined by comparing the tapped density to the bulk density using the equation:

Hausner’s ratio = Tapped density / Bulk density.

*Physicochemical properties of discs*

Each disc contained 300 mg of MH microspheres. The discs were round and flat with an average diameter of 8 ± 0.1 mm and discs were compressed with a constant compression force (3.5 tones). Weight variation was determined on discs as per the requirement of discs with average weight < 300 ± 0.005 mg. Hardness of the discs was performed on six discs using Erweka, hardness tester (Germany).

10 discs were placed in the plastic chamber that revolved at 25 rpm, dropping the discs a distance of six inches with each revolution. Normally, a pre-weighed discs sample is placed in the friabilator (W_1_), which is then operated for 100 revolutions. The discs are then dusted and reweighed (W_2_). Conventional compressed tablets that that lose than 0.5-1% of their weight are generally considered acceptable.

% Friability = W_2_-W_1_/W_1_ X 100

*Content uniformity*

 Content uniformity of discs was done by weighting the 3 discs and crushed with mortar and pestel, and then 50 mg of mixture were dissolved in 100 mL of 0.1 M HCl. This solution was filtered and the filtrate was diluted up with 0.1 M HCl and the drug concentration was measured spectrophotometrically at 205 nm.

*Evaluation of gastric-mucoadhesion properties (microparticles and discs)*

*Surface pH*

The surface pH of microparticles and the prepared discs was determined to evaluate the possible irritation to gastric mucosa. Microparticles and discs was allowed and swell with 50 mL of 0.1 M HCl (pH 1.2) and pH was measured at time intervals of 0, 1, 2, 4, 6 and 8 hours by using glass electrode in contact with microparticles and discs on pH meter (Corning pH meter 120, USA).

*Swelling study*

After weighting the microparticles and discs (W_1_), they were immersed in 0.1 M HCl (pH 1.2) at 37 °C. The weight of microparticles and discs was determined (W_2_) at time intervals of 0, 1, 2, 4, 6, 8 hours, the particles and discs was removed from solution and excess surface medium was removed carefully using the filter paper. The swelling index was calculated from the formula:

%Swelling index = (W_2_-W_1_)/W_1_ x 100

*In-vitro gastroretention time*

The gastroretention time studies were carried to *ex-vivo* mucoadhesion test. A segment of rat stomach mucosa, 3 cm long, was glued to the surface of a glass slide, vertically attached to the apparatus. The microparticles and discs was applied on the rat stomach mucosa which was fixed on the glass slide with cyanoacrylate glue and allowed to remove up and down so that was completely immersed in the 0.1 m HCl (pH 1.2). The slides were allowed to reciprocate in the medium until the microparticles and discs got detached or eroded from the mucosa. The experiment was carried out on three discs.

*Adhesion strength measurement*

**Figure 2:**
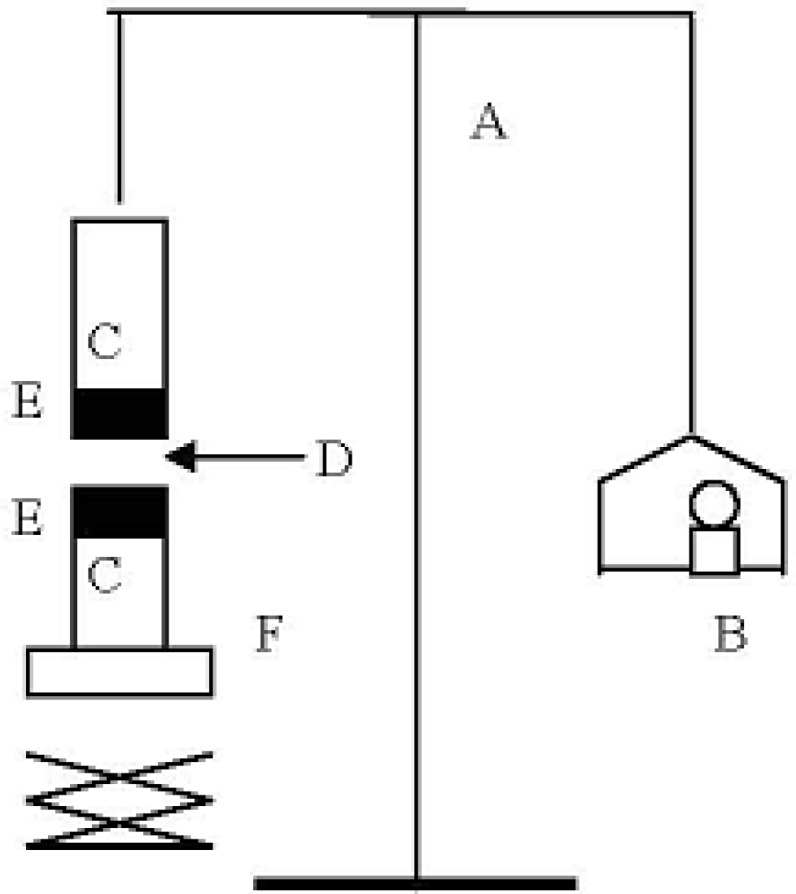
Bioadhesive force measuring device: (A) modified balance; (B) Weights; (C) glass vial; (D) MH discs; (E) rat tissue; (F) height-adjustable pan

The mucoadhesive forces of microparticles and discs were determined by means of the mucoadhesive force-measuring device shown in Figure 2 and according to the previously reported methods (11), using tissue cut from mucosal area abdominal of rat (hairless or cut the hair). The pieces of stomach were stored frozen in phosphate buffer pH 7.4, thawed to room temperature before use (12). At the time of testing, a section of stomach was secured to the upper glass vial (C) using a cyanoacrylate adhesive (E). The diameter of each exposed mucosal membrane was 1.5 cm. 

The vials were equilibrated and maintained at 37 °C for 10 min. Next, one vial with a section of tissue (E) was connected to the balance (A) and the other vial was fixed on a height-adjustable pan (F). To exposed tissue on this vial, a constant amount of discs (D) was applied. The height of the vial was adjusted so that the discs could adhere to the mucosal tissues of both vials. Immediately, a constant force of 0.5 N was applied for 2 minutes to ensure intimate contact between the tissues and the samples. The vial was then moved upwards at constant speed, it was connected to the balance. Weights were added at a constant rate to the pan on the other side of the modified balance of the used device until the two vials were separated. During measurement, 150 μL of stimulated gastric solution (0.1 M HCl, pH 1.2) was evenly spread onto the surface of the test membrane. The bioadhesive force, expressed as the detachment stress in g/cm^2^, was determined from the minimal weights that detached the tissues from the surface of each formulation using the following equation (12).

Detachment Stress gcm2=mA

Where *m* is the weight added to the balance in grams and *A* is the area of tissue exposed. Measurements were repeated thrice for each of the discs. All the above three experiments were conducted in triplicates (Figure 2).

*Histopathological Evaluation of gastric mucosa*

Histopathological evaluation of tissue incubated in 0.1 M HCl, pH 1.2, was compared with that treated with gastric mucoadhesive discs for 8 h. The tissue was fixed with 10% formalin, routinely processed, and embedded in paraffin. Paraffin sections were cut on glass slides and stained with hematoxylin and eosin. A pathologist blinded to the study to detect any damage to tissue and examined sections on light microscope (12).

*In-vitro dissolution analysis*

*In-vitro* dissolution studies were carried out on the microsphere at 37 °C ± (0.5 °C) at 100 rpm with USP dissolution apparatus II; 300-mg MH microspheres/discs was place into the dissolution apparatus.

The *in-vitro* dissolution studies were performed at pH 1.2, *i.e*., simulated gastric fluid pH. An accurately weight sample was responded in dissolution media consisting 900 mL of 0.1 N (pH 1.2) HCl and the dissolution was done for 8 h. The sample (5 mL) was withdrawn at each 0.25, 0.5, 1, 2, 3, 4, 5, 6, 8 hours interval and replaced with the same volume of test medium and the withdrawn samples were diluted if required and then estimated for MH concentration at 205 nm spectrophotometrically (UV-160, Shimadzu, Japan). Finally, each experiment was repeated three times (*n* = 3).

Kinetic parameters were also obtained by mathematical processing of drug release data. Evaluation of the influence of formulation variables on release rata constant *k* values, obtained for different groups of microsphere preparation.

In order to have a better comparison between different formulations dissolution efficiency (DE), t_50_% (dissolution time for 50% fraction of drug); and difference factor, f_1_ (used to compare multipoint dissolution profiles) were calculated (13). DE is defined as the area under the dissolution curve up to a certain time, *t*, expressed as a percentage of the area of the rectangle arising from 100% dissolution in the same time. The areas under the curve (AUC) were calculated for each dissolution profile by the trapezoidal rule. DE can be calculated by the following:

$\mathrm{DE}=\int y\frac{\mathrm{dt}}{100t}$

Where *y *is the drug percent dissolved at time *t*. All dissolution efficiencies were obtained with *t *equal to 480 min. The *in-vitro* release profiles of different microparticle formulations were compared with disc formulations using difference factor (f_1_), as defined by (13):

f_1_= {[Σ _t=1_^n^ |R_t_-T_t_|] / [Σ _t=1_^n ^R_t_]} ×100

Where *n *is the number of time points at which % dissolved was determined, *R*_t _is the % dissolved of one formulation at a given time point and *T*_t _is the % dissolved of the formulation to be compared at the same time point. The difference factor fits the result between 0 and 15 when the test and reference profiles are identical, and approaches above 15 as the dissimilarity increases. 

## Results and Discussion

*Physicochemical Properties of microparticles/discs*

Physicochemical characteristics of the microparticles are shown in Table 2. Microparticles were formed after a series of steps such as emulsion solvent evaporation (ESE). Each step of microsphere preparation was keenly observed to understand the effect of polymers (CP:EC) ratio on the particle size, total entrapment and release profiles of the drug-loaded microspheres. The polymers ratio was varied by maintaining the amounts of drug, solvent (O_1_), organic solvent (O_2_) and emulsifier agent constant in all preparations, while changing the amount of EC polymer (Table 1). The results of the effect of the polymers ratio on production yield, drug loading efficiency and mean particle size are shown in Table 2. 

**Table 2:** Effect of polymers ratio on the content, production yield and particle size of Metformin Hydrochloride microparticles

**Formulations**	**Carbomer 934 :** **EC** **ratio**	**Production** **yield** **(%±SD)**	**Theoretical** **drug** **Content** **(%)**	**Mean drug** **Entrapped (%±SD)**	**Drug loading** **efficiency** **(%±SD)**	**Mean particle** **Size** **(µm±SD)**
F_1_	1:2	89.64 ± 3.54	42.55	33.47 ± 1.78	78.66 ± 4.19	794.33 ± 25.11
F_2_	1:3	98.80 ± 6.07	33.33	32.81 ± 2.49	98.44 ± 6.98	933.25 ± 10.47
F_3_	1:4	85.74 ± 2.48	30.77	25.19 ± 2.37	81.87 ± 7.73	1071.52 ± 10.30

As the ratio of polymers (CP:EC) increases (1:2 to 1:4 ratio) the amount of free drug lost (Table 2), so that at the ratio of polymers (F_2_, 1:3 ratio) the amount of drug entrapment was 32.81 ± 2.49%, which was close to the theoretical value (37.04%). The Table also shows the lowest of polymer amount (EC) was in F_1_, (450 mg), however this formulation did not show the highest loading efficiency (78.66%). The particle analysis of microspheres prepared is shown in Table 2. The Table shows that an increase in polymers ratio from 1:2 to 1:4 result in a significant effect on the mean particle size of microparticles (for F_1_, F_2_ and F_3_, 794.33, 933.25 and 1071.52 µm, respectively). The analysis of data showed that all obtained microparticles followed a log-probability distribution. Mean particle size of original MH, EC and CP was 549.54 ± 10.72 μm, 489.78 ± 4.68 μm and 10.23 ± 1.22 μm, respectively.

Important prerequisites for high encapsulation efficiencies by the o_1_/o_2_ method are: (a) the insolubility of the drug in the external phase from the internal phase, and (b) the fine dispersion of the drug solution into the organic polymer solution to form a o_1_ phase (14, 15).

 Ethanol was used as a solvent, which can dissolve drug and polymers. In the preparation process of the microparticles, evaporation of the solvent increased co-precipitation of the drug and the polymers in the droplets. In all of the microparticles prepared, the amount of drug entrapped in microparticles was lower than the theoretical value. This indicates that some free drug crystals were lost in the process of encapsulation.

Increasing the amount of polymer in the organic phase can increase the viscosity of the external phase of the primary emulsion and this in turn possibly induced highly sticky droplets in the early stages of the preparation process due to the semi-solid polymer, and resulted in the droplets gathering together and hence drug don’t immigrate to the external phase. 

This is the main reason for the high drug entrapment and loading efficiency observed for polymers ratio (1:3), it is optimum polymers ratio between all of formulations. Generally, increasing the EC polymer amount increased the production yield (Table 2). The reason for this increase in high polymers ratio could be due to a reduction in the diffusion rate of solvent from concentrated solutions (organic phase) into the external phase of emulsion.

The microparticles size depended on the rate of polymer solidification. Since the polymers deposition within the droplets occurs through the removal of the polymers solvent (ethanol), the partitioning rate of ethanol from emulsion to external phase could be the main factor controlling the deposition rate of the polymers.

This could be due to solubility of MH in ethanol, which is able to increase the viscosity of the internal phase significantly. Also, these formulations showed high drug entrapment and loading efficiency. The reason for the increased production yield at high ratio of polymers (F_2_, 1:3 ratio) could be due to decreased evaporation rate of solvent from the concentrated solutions into the external phase.

Angle of repose for microspheres was between 16.18° to 18.25°, thus indicating good flow property for microspheres (Table 3). The findings were supported by Carr’s (compressibility) index, which was < 20 indicating good flow characterizes (F_1_, F_2_). The Hausner ratio is correlated to the flowability of a powder or microspheres. A Hausner ratio greater than 1.25 is considered to be an indication of poor flowability (Table 3).

**Table 3:** Flowability Characteristics of microparticle Formulations

**Formulations**	**Polymers (CP:EC) ratio**	**Bulk Density** **(g/cm** ^3^ **± SD)**	**Tapped Density** **(g/cm** ^3^ **± SD)**	**Carr’s index (%±SD)**	**Hausner ratio** **(±SD)**	**Angle of repose** **(°θ ±SD)**
F_1_	1:2	0.316 ± 0.02	0.380 ± 0.02	16.66 ± 0.00	1.200 ± 0.00	18.25 ± 0.22
F_2_	1:3	0.360 ± 0.00	0.434 ± 0.00	17.11 ± 0.01	1.206 ± 0.01	16.36 ± 1.14
F_3_	1:4	0.374 ± 0.01	0.488 ± 0.01	23.34 ± 0.00	1.304 ± 0.00	16.18 ± 0.56

The data indicates that the friability was less than 1% in F´_1_ disc ensuring that the discs were mechanically stable.

According to Table 4, standard deviation for weight variation of discs (≈300 mg) was less than the percentage deviation allowed under weight variation test for tablet (7.5% percentage deviation are allowed for 130-324 mg tablet) (1).

**Table 4:** Characteristics of disc Formulations

**Formulations**	**Polymers** ** (CP:EC)** **ratio**	**Weight Variation** **(mg±SD)**	**Content Uniformity** **(% ± SD )**	**Hardness** **(N ± SD)**	**Friability (%)**
F´_1_	1:2	298±0.002	96.32 ± 0.62	24.28 ± 1.63	0.3
F´_2_	1:3	299±0.005	95.95 ± 0.20	23.58 ± 2.01	5
F´_3_	1:4	298±0.001	95.45 ± 0.45	22.29 ± 1.28	15

**Figure 3:**
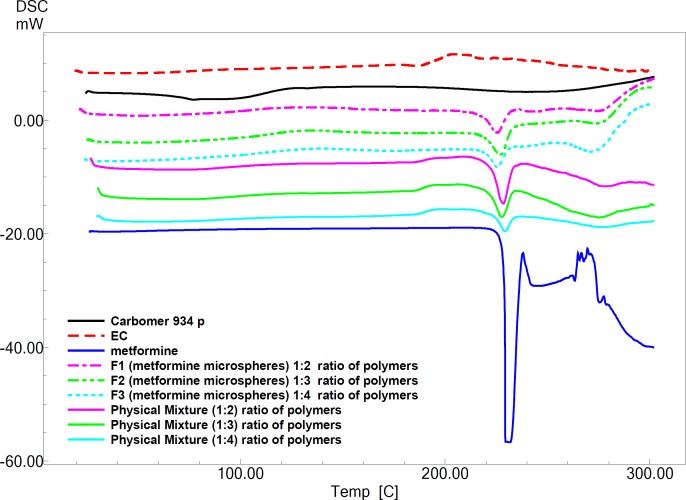
DSC thermogram of Carbomer 934 p; (EC) Ethylcellulose; metformin hydrochloride (MH); microspheres of F_1_, F_2_, F_3_ and physical mixture of F_1_, F_2_ and F_3_, respectively

Pure MH exhibits a sharp melting endotherm around 231.27 °C (Figure 3). It is obvious from thermograms that the DSC curves of physical mixtures of drug with polymers as well as the microparticle formulations are almost the same. This endotherm of the drug is present in most of the thermograms F_1_, F_2_ and F_3_ at 223.86 °C ، 220.27 °C and 221.41°C, respectively (Figure 3). The intensity of the drug fusion peak, however, for the microparticle formulations was lower than that of the pure drug and physical mixtures.

The DSC analysis of microspheres revealed negligible change in the melting point of MH indicating no modification or interaction between the drug and polymer.

The discs of all formulations had good appearance: 98.33-99.66% weight variation, 22.29-24.28 N hardness, and 0.3-15% friability. Hardness of discs was 3 ± 0.52 N.

The surface pH of all gastric-adhesive microparticles with in the range 1.032 to 1.173 but for discs in range 1.060 to 1.271 complies with referred value 1.2 in stomach (Table 5). 

**Table 5:** physicochemical characteristics of gastric-mucoadhesive microparticles and discs

**Residence on the mucus** **(%±SD)**	**Mucoadhesive** **strength (g/cm** ^2^ **±SD)**	**Swelling (%±SD)**	**pHsurface (±SD)**	**Formulations**
36.25 ± 5.30	-	138.22 ± 3.97	1.057 ± 0.03	F_1_
1.25 ± 1.76	-	133.71 ± 1.42	1.032 ± 0.00	F_2_
0.00 ± 0.00	-	95.77 ± 10.54	1.035 ± 0.01	F_3_
-	2.736 ± 0.24	90.16 ± 3.55	1.147 ± 0.01	F’_1_
-	1.758 ± 0.27	83.09 ± 2.24	1.162 ± 0.01	F’_2_
-	0.746 ± 0.05	83.09 ± 2.24	1.166 ± 0.01	F’_3_

*All of results is related to 8th h

**Figure 4:**
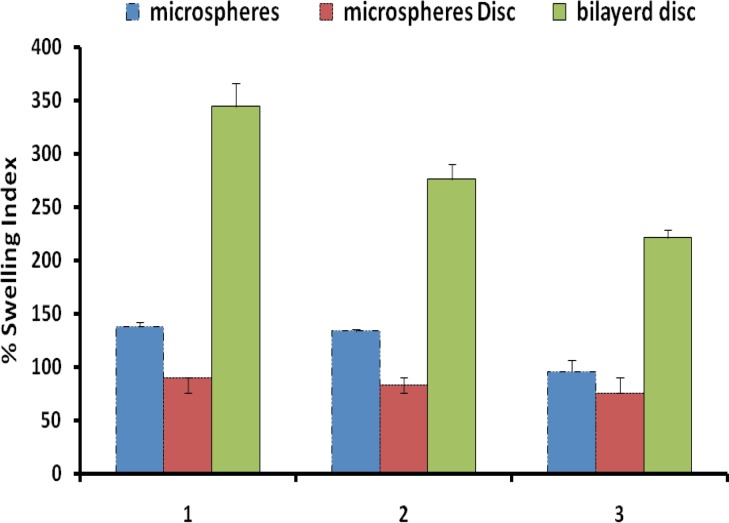
Comparative swelling indices of different formulations in 0.1N HCl after 8 h

Figure 4 shows the percentage swelling of different microparticles/discs formulations in 0.1 N HCl after 8 hours. The results revealed that all microsphere formulations swelled rapidly when immersed in 0.1 M HCl (pH 1.2). The percent swelling of different microparticle formulations was found to follow the rank order 101.43 ± 25.02% (F1), 107.81 ± 7.18% (F2) and 84.62 ± 6.60% (F3), respectively, after 1 h for microsphere prepared. After 8 h of incubation percent swelling was observed to be 138.22 ± 3.97% (F1), 133.71 ± 1.42% (F2) and 95.77 ± 10.54% (F3), respectively (Table 5). Discs showed significantly less (p < 0.05) swelling as comparison to microparticles. The effect of EC concentration on the swelling index in the microparticles/discs after 8 hours wetting were investigated. 

All of the formulations were near to pH 1.2 and hence expected to be non-irritant to gastric mucosa.

It is reported that adhesive properties and cohesiveness of mucoadhesive polymers are generally affected by their swelling behavior (16, 17).

It was observed that discs swelled slowly and produced higher mucoadhesive strength. Mucoadhesive microparticles are anticipated to take up water from the underlying mucosal, tissue by absorbing, swelling, and capillary effects, leading to considerable stronger adhesion. This is perhaps because slow swelling avoids the formation of over hydrated structure that loses its mucoadhesive properties before reaching the target (16). On the other hand, the highest swelling observed in microspheres could be due to its high amount of carbomer 934p (1:2 ratio) at pH 1.2, which is capable of absorbing a high amount of water (16). Swelling of discs involves the absorption of a liquid resulting in an increase in weight and volume. Liquid uptake by the particle may be due to saturation of capillary spaces within the particles or hydration of microparticles/discs. The liquid enters the particles through pores and bind to large particles, breaking the hydrogen bond and resulting in the swelling of microparticles/discs. Water uptake by cross-linked hydrogels (carbomer 934p) may occur initially through metastable pores and as swelling proceeds, mechanism is replaced by diffusion (18). Swelling depends on the polymer concentration, the ionic strength, and the presence of water. In the case of microparticles suggests that incorporation of water-insoluble polymer like EC leads to a rigid structure (1,19).

The degree of swelling is related to both drug release kinetics and mucoadhesiveness. Rapidly swollen discs are mucoadhesive. Excessive swelling again leads to reduced mucoadhesiveness, because water molecules bind the polymer carboxyl groups required for adhesion (20). 

The *in-vitro* residence time with rat stomach mucosa in simulated gastric (pH 1.2) varied for microparticles from 0 to 480 min. Microparticles showed highest mucoadesion in this study, and did not dissolve in 0.1 M HCl for about 8 h. For F_1_ formulation, percentage of microparticles remaining was 36.25%. (number of microsphers at zero time was considered 100% and after passed the time until 8 hours some microspheres moved on the mucos In contrary, F_2_ and F_3 _showed relatively lower retentive than F_1_ formulation.

**Figure 5:**
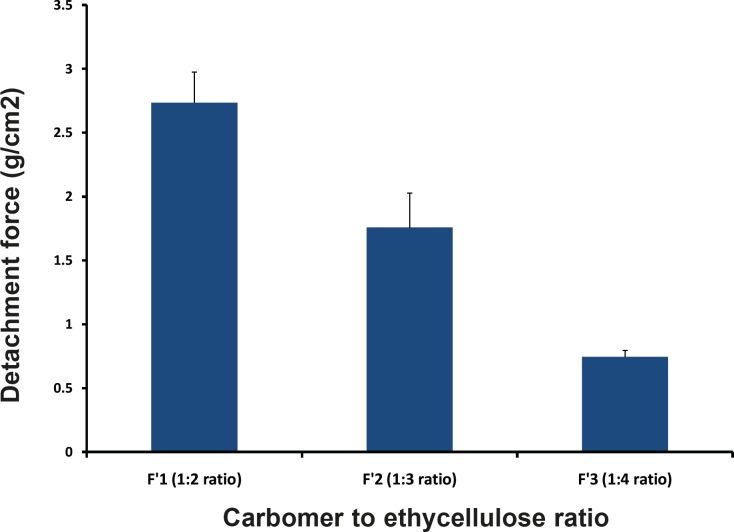
Comparative swelling indices of different formulations in 0.1N HCl after 8 h.

It was observed that the effect of concentration of EC on the *in-vitro* residence time was significant, with microparticles containing high proportion of EC eroding rapidly and giving short residence time (F´3, 1:4 ratio). F´_1_, F´_2_ and F´_3_ Formulations containing the same levels of CP but different levels of EC demonstrated that decrease in the amount of residence time, respectively. Thus, EC had a negative effect on *in-vitro* residence time. A similar effect has been demonstrated in the buccal patch of sumatriptan succinate by Shidhaye *et al *(12).

*In-vitro *bioadhesive strength study was performed and the results are shown in the Table 5 and Figure5. On the modified physical balance and measure the force (g) required detaching the disc. The bioadhesion characteristics were affected by the concentration of the bioadhesive polymers. Increase in concentration of polymer increases bioadhesive strength of formulation. The formulations (F´_1_, F´_2_ and F´3) with CP and EC showed the bioadhesive strengths of 2.74, 1.76 and 0.746 g/cm^2^, respectively. F_1_ Formulation containing 1:2 ratio (CP:EC) showed the highest mucoadhesivity (2.736 ± 0.24 g/cm^2^). 

**Figure 6:**
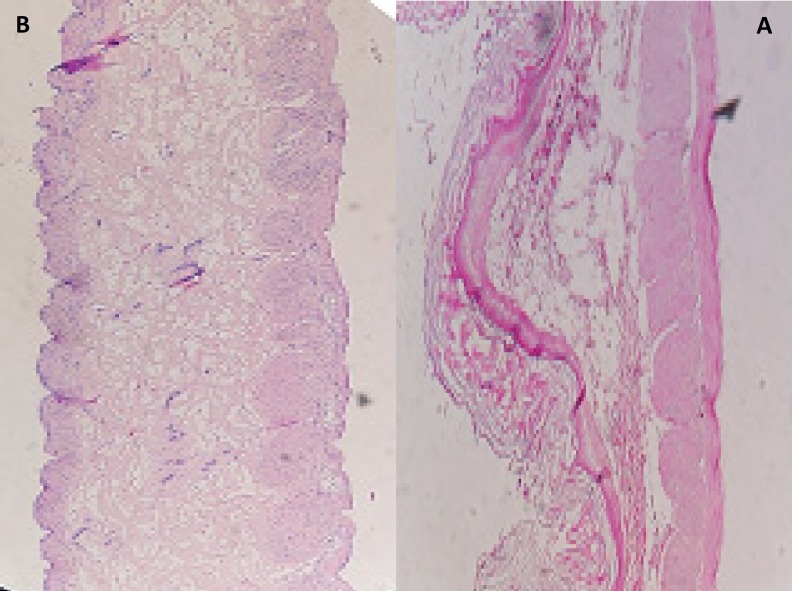
Histopathological evaluation of sections of rat gastric mucosa (A) un-treated (B) treated with microparticles discs containing MH (magnitude X).

The microscopic observations indicated that the microparticles had no significant effect on the microscopic structure of mucosa. As shown in Figure 6, no-cell necrosis was observed. 

Poor mucoadhesion of EC polymer at acidic pH may be due to its non-ionic nature possessing low hydrogen bonding capability with mucus glycoproteins, while excellent mucoadhesion of CP was from the electrostatic attraction between CP (anionic in nature) and mucin (anionic in nature). Although microparticles containing CP polymer had negative charge repulsion with mucus, numerous hydrophilic functional groups such as carboxyl groups in carbopol molecules could form hydrogen bonds with mucus molecules, thus producing some adhesive force of this polymer (CP). The greater mucoadhesivity of F_1_ microparticles were due to anionic nature of the polymer (CP) which is desirable characteristics of adhesion to the mucus layer. Adhesion of polymer with the mucus membrane is mediate by hydration in the case of hydrophilic polymer. Upon hydration these polymers becomes sticky and adhere to mucus membrane. 

*In-vitro release studies*

**Figure 7:**
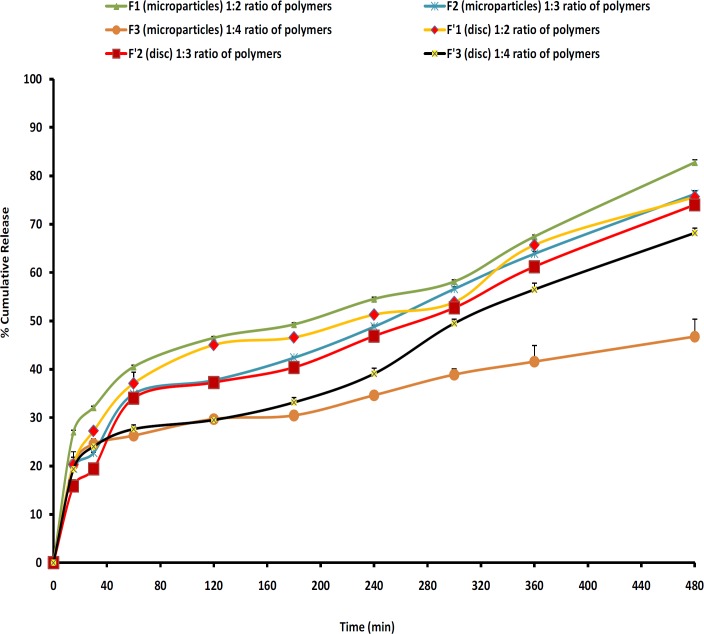
Cumulative percent release of MH from microparticles and discs prepared with different polymers ratios

The release profiles for all microparticles are illustrated in Figure 7. In order to have better comparison between the dissolution profiles, dissolution efficiency, t_50%_, Q_2_ and Q_8_ were calculated and the results showed that microparticles with high loading efficiency or high drug entrapment showed lowest burst effect (F2). Figure 7 and Table 6 show that the initial drug releases for the microparticle formulations are high. Rel_2_ was for F_1_, F_2_ and F_3_ as 46.48%, 37.73% and 44.23%, respectively. As more drugs are released from the microparticles, more channels and pores are probably produced, contributing to faster drug release rates. Discs showed lower burst release and F_3_ resulted in the lowest burst release (29.5%) in comparison with other formulations and the percentage of burst release reduced as the increasing of polymers ratio (F_3_, 1:4). F_2_ showed the highest production yield (98.80%) and loading efficiency (88.58%). 

Cellular membrane was intact and no damage was observed to the treated rat stomach mucosa. Thus, formulation containing microparticles appeared to be safe with respect to oral administration (Figure7).

**Table 6:** Comparison of various release characteristics of MH from different microsphere formulations, discs and commercial^® ^tablet

**Formulations**	**Polymers** **(CP:EC)** **ratio**	**Rel**_2 _^a^ **(%)**	^b^ **Rel**_8_ **(%)**	**DE** ^c^	^d^ **t**_50%_ **(h)**	^e^ **f**_1_
F_1_	1:2	46.48±0.27	82.76±0.5	55.19	3.1	7.78
F_2_	1:3	37.73±0.25	76.25±0.67	50.18	4.1	4.64
F_3_	1:4	44.23±1.32	69.62±1.32	51.52	4	20.56
F’_1_	1:2	45.02±0.73	75.62±0.65	51.78	4	8.44
F’_2_	1:3	37.25±0.90	73.98±1.07	48.21	4.4	4.87
F’_3_	1:4	29.5±0.78	68.16±1.05	42.32	5	25.88

^a^ Rel2 = amount of drug release after 2 h; ^b^ Rel_8_ = amount of drug release after 8 h; ^c^DE = dissolution efficiency; ^d^t 50% = dissolution time for 50% fractions; ^e^ f1 = Differential factor [formulations are compared with together (F_1_ with F’_1_, …, F_3_ with F’_3_)].

The reason for the burst release could be due to the presence of some MH particles close to the surface of the microspheres. When particles are prepared by the o_1_/o_2_ method, water-soluble drugs don’t have a tendency to migrate to the non-polar medium, thereby concentrating at the surface of the microspheres and inducing the burst effect (20). Moreover, the burst release could also be explained by the imperfect encapsulation of the drug inside microparticles, as a result of the unstable nature of the emulsion droplets during the solvent removal step. This potential instability may cause a part of the loaded drug to relocate at the microparticle surface, thereby being rapidly released (19). 

Figure 7 also shows that in most cases a biphasic dissolution pattern was observed. It can be supposed that the first portion of the curves is due to MH dissolution, which starts immediately after the beginning of the test for the portion of drug very close to the surface of microspheres. After such a phase, two phenomena can combine to enhance the diffusion of the remaining dispersed drug into the bulk phase as well as the formation of pores within the matrix due to the initial drug dissolution which enhances the permeability of the polymers to the drug (19).

EC has a low permeability to drug which results from its high intermolecular attraction. The pores present in EC polymer acts as a channeling agent for the entrance of the liquid medium through the microparticles wall, causing it to swell. Hydrogen bonding between the hydroxyl groups of the carboxylic moiety and the carbonyl oxygen of ester group increases the degree of solidity of the polymer and decreases its porosity and permeability. Thus, by varying the ratio of polymers (CP:EC) in the MH microparticles, the rate of release of MH can be controlled.

**Table 7:** Fitting parameters of the *in-vitro* release data to various release kinetics models

**Formulations**	**Order**	**MPE%**	**RSQ**	**Slope**	**intercept**	**K**
F_1_	Peppas	1.74	0.992	0.25	0.25	0.1391
F_2_	Peppas	4.46	0.963	0.329	-0.219	0.0012
F_3_	Peppas	3.46	0.950	0.189	-2.529	0.0797
F´_1_	Peppas	4.69	0.973	0.318	2.389-	0.0918
F´_2_	Higuchi	6.3	0.968	0.03	0.042	0.0304
F´_3_	Linear-probability	5.58	0.981	0.003	0.849-	0.0027

Comparing the drug release from microparticles prepared by EC and CP polymers shows that the release of drug from microparticles (t_50% _= 3.1-4 h) slower than the release of drug from discs (t_50% _= 4-5 h) (p > 0.05). However, a no-significant difference was observed between the percentages of drug released at 8 hours (Rel_8_) between microparticles and discs (p > 0.05). The highest drug release at 8^th^ hours (pH 1.2) with F_1_ microparticles (82.76%) compared to other microspheres may be due to the lower concentration of EC (1:2 ratio). The difference factor test showed that microparticle formulations match the release profile of discs and there was not a significant difference between these dissolution profiles (except F_3_ with F’_3_). 

A high correlation was observed between the Peppas and linear-probability order model (Table 7). The data obtained were also put in the Korsemeyer-Peppas model in order to find out the n value, which describes the drug release mechanism (22). The n value of microparticles of different polymers ratio was between 0 < n< 0.5, indicating that the mechanism of the drug release was diffusion controlled (Table 7). 

## Conclusion

The present study has been satisfactorily attempted to formulate a mucoadhesive microparticulate system of an antidiabetic drug like MH for oral administration with a view of combination of two polymers (CP and EC). % entrapment efficiency was higher for F_2_ microparticles than other formulations. While practical yield obtained was higher for F_2_ microparticles. The particle size analysis revealed that all formulations gave particles in the range of 794.33-1071.52 μm which is suitable for oral administration of formulation. Increase in the mucoadhesive polymer led to increase in mucoadhesion and degree of swelling. However, Carbopol showed higher mucoadhesion and swelling degree than EC. As the concentration of mucoadhesive polymer increased, the drug releases also idecreased proportionally, because it produced hydrogel on the mucus. From all the parameters studied, it can be concluded that microparticles and discs do not have significant difference together (p > 0.05). MH microparticles are better gastric-adhesive delivery system for the formulation of MH for gastro-intestinal administration. Thus, the formulated microspheres seem to be a potential candidate as oral controlled drug delivery system for diabetes therapy.
